# Hantavirus cardiopulmonary syndrome successfully treated with high-volume hemofiltration

**DOI:** 10.5935/0103-507X.20160032

**Published:** 2016

**Authors:** Guillermo Bugedo, Jorge Florez, Marcela Ferres, Eric Roessler, Alejandro Bruhn

**Affiliations:** 1 Departamento de Medicina Intensiva, Escuela de Medicina, Pontificia Universidad Catolica de Chile - Santiago, Chile.; 2 Departamento de Enfermedades Infecciosas e Inmunologia Pediátrica, Escuela de Medicina, Pontificia Universidad Catolica de Chile - Santiago, Chile.; 3 Departamento de Nefrología, Escuela de Medicina, Pontificia Universidad Catolica de Chile - Santiago, Chile.

**Keywords:** Sepsis, Hantavirus pulmonary syndrome/therapy, Hemofiltration/therapeutic use, Case reports

## Abstract

Hantavirus cardiopulmonary syndrome has a high mortality rate, and early connection to extracorporeal membrane oxygenation has been suggested to improve outcomes. We report the case of a patient with demonstrated Hantavirus cardiopulmonary syndrome and refractory shock who fulfilled the criteria for extracorporeal membrane oxygenation and responded successfully to high volume continuous hemofiltration. The implementation of high volume continuous hemofiltration along with protective ventilation reversed the shock within a few hours and may have prompted recovery. In patients with Hantavirus cardiopulmonary syndrome, a short course of high volume continuous hemofiltration may help differentiate patients who can be treated with conventional intensive care unit management from those who will require more complex therapies, such as extracorporeal membrane oxygenation.

## Abstract (pt)

A síndrome cardiopulmonar por hantavírus tem elevada taxa de mortalidade. Sugere-se que uma conexão precoce com oxigenação por membrana extracorpórea melhore os resultados. Relatamos o caso de uma paciente que apresentou síndrome cardiopulmonar por hantavírus e choque refratário, que preenchia os critérios para oxigenação por membrana extracorpórea e que teve resposta satisfatória com uso de hemofiltração contínua de alto volume. A implantação de hemofiltração contínua de alto volume, juntamente da ventilação protetora, reverteu o choque dentro de poucas horas e pode ter levado à recuperação. Em pacientes com síndrome cardiopulmonar por hantavírus, um curso rápido de hemofiltração contínua de alto volume pode ajudar a diferenciar pacientes que podem ser tratados com cuidados convencionais da unidade de terapia intensiva dos que necessitarão de terapias mais complexas, como oxigenação por membrana extracorpórea.

## INTRODUCTION

Hantavirus cardiopulmonary syndrome (HCPS), also known as Hantavirus pulmonary syndrome (HPS), has a 35% overall case fatality rate.^(1)^ There are no specific antivirals, vaccines, or immunotherapeutic agents for HCPS, and treatment is mainly supportive and symptomatic.^(2)^ Ribavirin is an antiviral agent that did not yield any significant benefit in clinical outcomes when used to treat patients during the cardiopulmonary phase of the disease.^(3)^ A recent trial of high-dose methylprednisolone (16mg/kg/day) in patients with confirmed or suspected HCPS, attempting to modulate the immune response responsible for the catastrophic outcome, showed no significant clinical benefit.^(1)^

Patients with HCPS and refractory shock have a particularly high mortality rate, and early connection to extracorporeal membrane oxygenation (ECMO) has been suggested to improve outcomes.^(4,5)^ However, despite an overall survival of 66%, complications from percutaneous cannulation and bleeding are frequent with ECMO. There has also been no prospective trial comparing ECMO with a more conservative approach that incorporates recent advances in critical care management.^(6)^ As part of our protocol for managing patients with septic shock, high-volume continuous hemofiltration (HVHF) is frequently used and may play a role in decreasing mortality in patients with refractory septic shock at our institution.^(7-9)^

We report the case of a patient with demonstrated HCPS and refractory shock who was successfully treated with HVHF and Hantavirus hyperimmune plasma, the latter as part of a compassionate treatment national protocol.^(10)^ A short course of HVHF may help to differentiate patients who can be treated with conventional intensive care unit (ICU) management from those who will require more complex therapies, such as extracorporeal membrane support.

## CASE REPORT

A 30-year-old female patient was admitted to our emergency department in February 2013 with a six-day history of malaise and headache followed by fever. Three days before admission, she was seen in another hospital and sent home with Levofloxacin for suspicion of sinusitis. When symptoms worsened and she developed progressive dyspnea, she visited our emergency department.

**Figure 1:**
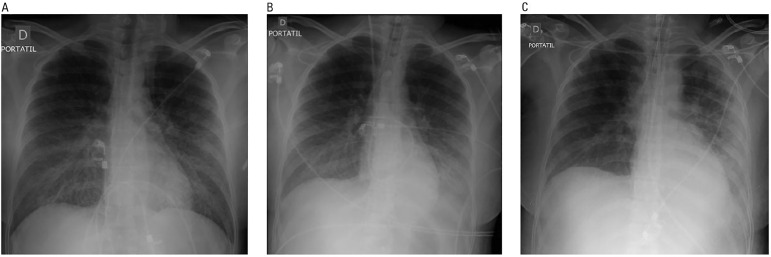
Chest-X-ray on A) admission (left), B) the first day (center) and C) the 6^th^ day (right) showing bilateral infiltrates suggesting acute respiratory distress syndrome.

On admission, she had severe dyspnea and tachycardia. Laboratory results indicated a lactate level of 4.2mmol/L, C-reactive protein of 7.7mg/dL (normal value < 0.5), platelet count of 34,000/mm^3^, hematocrit of 47.9%, and lactate dehydrogenase of 406U/L. Diffuse bilateral perihilar infiltrates were present on chest radiography (Figure 1). Troponin-T and creatine-kinase MB were within normal limits. A blood smear showed both neutrophilia and lymphocytosis, including lymphocytes with immunoblastic morphologic features. Because she had recently travelled to an area with a high prevalence of Hantavirus, a rapid test was ordered on admission, and the results were positive.

After initial resuscitation with normal saline, she was transferred to the ICU. Subsequently, due to poor oxygenation and severe alterations in clinical perfusion, the patient was intubated, and protective ventilation was initiated according to our protocol.

Despite administration of hydrocortisone 100mg and aggressive volume expansion with normal saline and albumin, within a few hours, the patient required high dose norepinephrine (0.7µg/kg/min). Peripheral perfusion was also severely altered (lactate levels 5.9mmol/L). Echocardiography showed depressed left ventricular function, with an estimated ejection fraction (Simpson) of 40%, in the presence of severe tachycardia (Table 1). A pulmonary artery catheter showed a cardiac index of 2.8L/min/m^2^ and a stroke volume index of 17.5mL/min/m^2^, confirming the diagnosis of cardiopulmonary syndrome secondary to Hantavirus infection.

**Table 1:** Time course of hemodynamic, clinical and laboratory variables immediately before and after starting high volume hemofiltration

**Time**	**Baseline**	**3 hours**	**6 hours**	**12 hours**	**24 hours**	**48 hours**	**72 hours**
Cardiac index (L/min/m^2^)	2.8		3.1	3.2	3.4	3.9	2.7
PWP (mmHg)	20		15	15	16	8	18
CVP (mmHg)	14		10	7	12	7	14
PAP (mmHg)	36/26		26/14	27/15	27/10	24/9	27/15
SVI (mL/min/m^2^)	17.5		24.8	25.6	25.5	35.1	27.8
Heart rate	160	129	125	125	133	111	97
Temperature (ºC)	38.2	36.4	36.2	36.3	36.2	36.5	36.6
NE dose	0.7	0.42	0.16	0.11	0.04	0.1	-
Fluid balance* (L)	3.6				3.9	4.8	0.5
PaO_2_ (mmHg)	81.2	66.7	82	86.5	88.6	115.5	89.9
PaCO_2_ (mmHg)	31	30	31.4	26.6	33.7	38.9	32
pH	7.28	7.31	7.41	7.45	7.44	7.36	7.44
HCO_3_ (mEq)	14.3	14.7	19.6	18	22.2	21.6	21
PaO_2_/FiO_2_ ratio	232	148	182	216	222	289	321
SatmvO_2_ (%)	74.3	62.7	67.6	63.4	66.9	79.5	54.9
Creatinine (mg/dL)	0.83				0.49	1.42	1.34
C-reactive protein (mg/dL)	10.6				14.8	7.3	
Lactate (mmol/L)	5.9	4.6	4.4	2.5	3.6	2.6	2.7
Albumin (g/dL)	2.6					2.7	2.7
Hematocrit (%)	40.6				32.1	29.7	28
WBC (/mm^3^)	23,800				28,000	25,100	18,500
Platelets (/mm^3^)	27,000				27,000	24,000	25,000

PWP - pulmonary artery wedge pressure; CVP - central venous pressure; PAP - pulmonary artery pressure; SVI - stroke volume index; NE dose - norepinephrine dose (µg/kg/min); PaO_2_ - arterial oxygen tension; PaCO_2_ - arterial carbon dioxide tension; HCO_3_ - bicarbonate; PaO_2_/FiO_2_ - ratio of arterial oxygen tension to inspired oxygen fraction; SatmvO_2_ - mixed venous oxygen saturation; WBC - white blood cells.

* Accumulated fluid balance during the previous 24 hours.

At that time, the ratio of arterial oxygen tension to inspired oxygen fraction (PaO_2_:FiO_2_ ratio) was approximately 200. However, due to severe cardiac dysfunction and high norepinephrine requirements, we decided to implement a trial of 6 hours of HVHF before deciding to initiate ECMO.

A 13.5 double-lumen catheter was inserted into the right femoral vein, and HVHF was initiated at 100mL/kg/h. Three hours after starting HVHF, norepinephrine levels were reduced by half, with a significant improvement in clinical perfusion. At that time, the patient received Hantavirus hyperimmune plasma (5,000U/Kg) under a compassionate national treatment protocol.^(10)^ Lactate levels decreased from 5.9 to 2.5mmol/L 12 hours after the initiation of HVHF and eventually normalized a few days later. Two days later, repeat echocardiography revealed an improvement in ejection fraction to 65% and an increase in stroke volume index to 35mL/min/m^2^.

The patient continued to improve clinically, although she developed hyperactive delirium, which was successfully managed with quetiapine and dexmedetomidine. The patient was finally extubated 10 days after admission.

Echocardiography on day 14 showed normal systolic function, with no evidence of pulmonary hypertension or cardiac chamber enlargement. On day 16 after admission, she was discharged from the ICU and was sent home a few days later. Two years later, the patient is living a normal life.

## DISCUSSION

We describe a patient with HCPS who presented with respiratory failure and severe cardiovascular dysfunction, fulfilling the traditional criteria for ECMO. The implementation of HVHF may have helped to reverse shock within a few hours and, together with conventional critical care management, may have prompted recovery.^(6-8,11,12)^

Hantavirus cardiopulmonary syndrome has high mortality. Treatment is mainly supportive and symptomatic. Early connection to ECMO has been suggested to improve outcomes in patients with HCPS and refractory shock.^(5)^ In 1998, Crowley et al. identified several criteria for non-survival, which included refractory shock, lactate > 4.0mmol/L, severe hypoxia (PaO_2_:FiO_2_ ratio < 60), and cardiac arrest.^(4)^ More recently, Wernly et al. reported an overall survival of 66.6% in 51 patients who were treated with ECMO support. The patients, who had at least one of the previous criteria for non-survival, had a typical clinical presentation consistent with HCPS and a cardiac index that rapidly dropped to < 2.0L/min/m2 despite maximum inotropic support.^(5)^ However, to date, there has been no prospective trial comparing ECMO with a more conservative approach that incorporates recent advances in critical care management, including protective ventilation and HVHF.^(7,8,11,12)^ Moreover, ECMO is associated with complications derived from vessel cannulation, frequent bleeding, and high costs.^(5)^

The symptoms and severity of HCPS are mainly due to increased capillary permeability following activation of the innate and adaptive immune response rather than direct virus-induced cellular damage. This is likely the most important physiological feature responsible for the massive leakage of plasma into alveoli, with resultant pulmonary edema, in Andes virus (ANDV) infection. In experimental and some observational trials, HVHF has been shown to remove excess inflammatory mediators and to improve cardiopulmonary function in refractory septic shock.^(7,13)^ However, a large randomized trial of 140 critically ill patients with septic shock and acute kidney injury did not show a benefit of HVHF in improving either hemodynamic profile or organ function at a dose of 70mL/kg/h compared with 35mL/kg/h.^(14)^

In the setting of HCPS, Seitsonen et al. reported two cases caused by Puumala virus infection that rapidly resolved after initiation of corticosteroid treatment combined with continuous veno-venous hemo-diafiltration.^(15)^ As the use of steroids has not been shown to provide significant clinical benefit in a number of patients,^(1)^ these cases reinforce the potential role that HVHF may play as an alternative way of modulating the inflammatory response in refractory shock in the context of HCPS before proceeding to ECMO.

Our patient fulfilled the criteria for ECMO due to the presence of refractory shock despite high doses of norepinephrine, hyperlactatemia and tissue hypoperfusion. However, hypoxemia was moderate, and the hemodynamic profile resembled that of severe septic shock. Therefore, based on our extensive experience with HVHF, we decided to initiate a trial of HVHF.^(7-9,16)^ The dramatic decrease in norepinephrine requirements soon after HVHF suggests that this therapy may have played a significant role in improving the patient's condition. However, whether this was due to the removal of inflammatory mediators, a decrease in fever or the normalization of pH remains unclear. If the patient had not responded well to HVHF therapy or presented with refractory hypoxemia, ECMO would have being started immediately.

We also cannot exclude the role of Hantavirus hyperimmune plasma in reversing shock. However, the temporal course suggests otherwise as the patient was already on HVHF and improving when the hyperimmune plasma was administered. The use of neutralizing antibodies produced by a DNA vaccine has been shown to protect against lethal ANDV infection in Syrian hamsters, an animal model of ANDV infection.^(17)^ A non-randomized pilot study in patients with ANDV infection was conducted in Chile. The results were promising, with a reduction in mortality from 32% to 14%, compared with the outcomes of patients from centers that did not participate in the study.^(10)^ However, future studies are needed to support a definitive recommendation regarding the use of immune plasma.

## CONCLUSION

In summary, we describe a patient with Hantavirus cardiopulmonary syndrome who presented with respiratory failure and severe cardiovascular dysfunction. Despite fulfilling traditional criteria for extracorporeal membrane oxygenation, the implementation of high-volume continuous hemofiltration was associated with rapid shock reversal and may have prompted recovery. In patients with Hantavirus cardiopulmonary syndrome and cardiovascular dysfunction, we suggest that a 6 to 12 hour trial of high volume continuous hemofiltration be attempted before proceeding to a more aggressive approach, such as extracorporeal membrane oxygenation. However, further clinical trials are required to support a definitive recommendation regarding the use of high volume continuous hemofiltration in these patients.
